# Sequenoscope: a modular tool for nanopore adaptive sequencing analytics and beyond

**DOI:** 10.1099/acmi.0.001059.v4

**Published:** 2026-05-11

**Authors:** Abdallah Meknas, Kyrylo Bessonov, Shannon H.C. Eagle, Christy-Lynn Peterson, James Robertson, Nicole Ricker, Tara Signorelli, John Nash, Aleisha Reimer

**Affiliations:** 1 National Microbiology Laboratory, Public Health Agency of Canada, Guelph, ON, Canada; 2 National Microbiology Laboratory, Public Health Agency of Canada, Winnipeg, MB, Canada; 3 Department of Pathobiology, Ontario Veterinary College, University of Guelph, Guelph, ON, Canada; 4 National Microbiology Laboratory, Public Health Agency of Canada, Toronto, ON, Canada

**Keywords:** adaptive sampling, metagenomics, method comparison, nanopore, sequencing, Oxford Nanopore Technologies (ONT), pipeline

## Abstract

This article presents Sequenoscope: a bioinformatics pipeline for analysing Oxford Nanopore Technologies (ONT) adaptive sampling sequencing data. Sequenoscope features three main modules: *filter_ONT* for filtering raw reads and creating a FASTQ file with a subset of reads for further analyses, *analyze* for generating sequencing and read mapping statistics against the provided reference taxon sequences and *plot* for interactive data summarization, comparison, and visualization between two datasets/test conditions. Here, we demonstrate the ability of the pipeline to analyse ONT adaptive sampling sequence data and provide examples of the outputs users can expect using data we generated. Adaptive sampling was performed on two ZymoBIOMICS Microbial Community DNA Standards, log-distributed (Cat# D6311) and even-distributed (Cat# D6306) formulations, with targeted depletions of *Listeria monocytogenes*. By comparing the test and control experimental data in FASTQ files from the sequencing runs, Sequenoscope showed that depletion of *L. monocytogenes* was successful by providing users with parameters to compare such as taxon coverage, read length and types of pore-level decisions made during sequencing. Although Sequenoscope was designed for ONT adaptive sampling data analysis, it supports short-read data from other sequencing platforms such as Illumina, allowing for the direct comparison of any two experimental conditions or cross-platform benchmarking.

**Impact Statement**

Adaptive sampling (AS) is a promising new technique available to Oxford Nanopore Technologies (ONT) users that offers targeted enrichment or depletion without any additional laboratory procedures. However, convenient tools for assessing the effectiveness of an AS experiment remain limited. Sequenoscope addresses this gap by offering outputs summarizing and visualizing key parameters from sequencing and genome mapping including taxon coverage depth and read length. While optimized for ONT long-read AS, Sequenoscope provides the flexibility to process Illumina short-read data, offering a versatile, cross-platform framework for comparative analysis across diverse experimental conditions. Its broad range of potential applications includes infectious disease diagnostics, pathogen surveillance, antimicrobial resistance studies, environmental DNA research, human genomics and others. Beyond these core application areas, Sequenoscope serves as a versatile resource for laboratories seeking user-friendly, accessible AS analytics in both clinical and research contexts.

## Data Summary

Basecalled sequence data (FASTQ files), generated from POD5 raw signal files, has been deposited under NCBI BioProject PRJNA1051081 with 12 associated SRA accessions (SRR27189470–SRR27189481). Sequenoscope is a Python 3 application available as PyPI, Conda and NextFlow packages under the Apache License, v2.0. The source code and documentation are available at the GitHub repository https://github.com/phac-nml/sequenoscope.

All supplementary material files (Tables S1–S30, Figs S1–S18 and the ONT sequencing summary file) are available via the Microbiology Society Figshare repository (DOI: https://doi.org/10.6084/m9.figshare.30898214)[1], covering analyses of all technical replicates performed in triplicate.

## Introduction

The introduction of Nanopore long-read sequencing by Oxford Nanopore Technologies (ONT) has brought significant advancements to genomic sequencing. ONT sequencing is able to generate longer DNA reads, offering a more contiguous view of genomes and enhancing the resolution of complex metagenomic samples. In metagenomics, DNA depletion strategies are crucial for optimizing the efficiency and specificity of genomic analysis by increasing the chances of sequencing low-abundance targets and/or removing contaminants [2]. This is particularly important in clinical samples, which often contain an excess of host DNA and abundant normal gut bacteria that can overshadow the pathogens of interest. Unlike host DNA, which can be depleted using specific laboratory methods, there are no direct methods to deplete normal gut bacteria. Conversely, targeted enrichment strategies such as amplicon sequencing or bait capture [3,4] can be used to enrich for taxa of interest; however, they add more time to laboratory procedures and usually require extensive research and development.

In 2016, the ReadUntil API was introduced, a novel ONT sequencing innovation facilitating real-time selection or rejection of sequencing reads which can reduce off-target sequencing [5]. In 2020, ONT announced integration of this API into their sequencing software MinKNOW named adaptive sampling (AS) [6]. AS selectively targets specific DNA sequences for enrichment or depletion, thereby enhancing the efficiency and precision of these sequencing technologies [7]. It uses voltage reversal to selectively sequence desired DNA reads, ‘kicking out’ unwanted reads and ‘continuing to sequence’ wanted reads. Conveniently, AS does not require additional laboratory preparation, making it a more efficient option for targeted sequencing. AS can greatly improve the efficiency of sequencing by spending more time sequencing on-target DNA.

AS using ONT sequencing devices has been applied in various contexts. For instance, the ReadFish open-source tool, which uses the ReadUntil API, demonstrated enrichment of specific human chromosomes and exons [8]. Other approaches used AS in MinKNOW to identify disease-causing variations [9] and detect antimicrobial resistance genes [10]. In metagenomics, this approach has been used to selectively deplete host DNA or enrich specific bacterial taxa, revealing pathogens within complex communities [11], thereby improving the feasibility of next-generation sequencing (NGS) for clinical diagnostics [12] and environmental analyses [13]. These examples demonstrate the impact of AS technology across genomics, diagnostics, and microbiology, offering a flexible approach to targeted sequencing that can be tailored to specific requirements. Despite the potential for AS, rapidly visualizing and evaluating its effectiveness remains a major hurdle. Many pipelines are not designed with AS in mind, limiting their ability to provide clear, informative run statistics or user-friendly reports.

We introduce **Sequenoscope**, a simple, modular command-line bioinformatics pipeline specifically designed to evaluate ONT AS sequence long-read data. Although Sequenoscope is compatible with both long- and short-read data, its primary focus remains on Nanopore (ONT) long reads. While support for short-read platforms (e.g. Illumina) is currently included to enhance tool versatility, this data type generates a limited set of outputs (as it lacks the real-time pore-level decision information) and has not yet been systematically benchmarked. Nevertheless, we performed a basic proof-of-principle validation using publicly available Illumina mock community short-read data. Sequenoscope compares distinct FASTQ files to assess the success or failure of enrichment or depletion strategies and offers data visualization via the Plotly graphing library [14]. The interactivity of Plotly allows users to explore results and easily interpret sequencing outcomes. Here, we describe the core functionalities of Sequenoscope and demonstrate its utility and versatility in analysing reference-mapped FASTQ files from AS workflows. Our goal is to provide a tool that facilitates data exploration, accelerates decision-making and delivers insights into the efficacy of targeted sequencing strategies for diverse genomic applications.

## Methods

### Bioinformatics pipeline overview

**Fig. 1.**
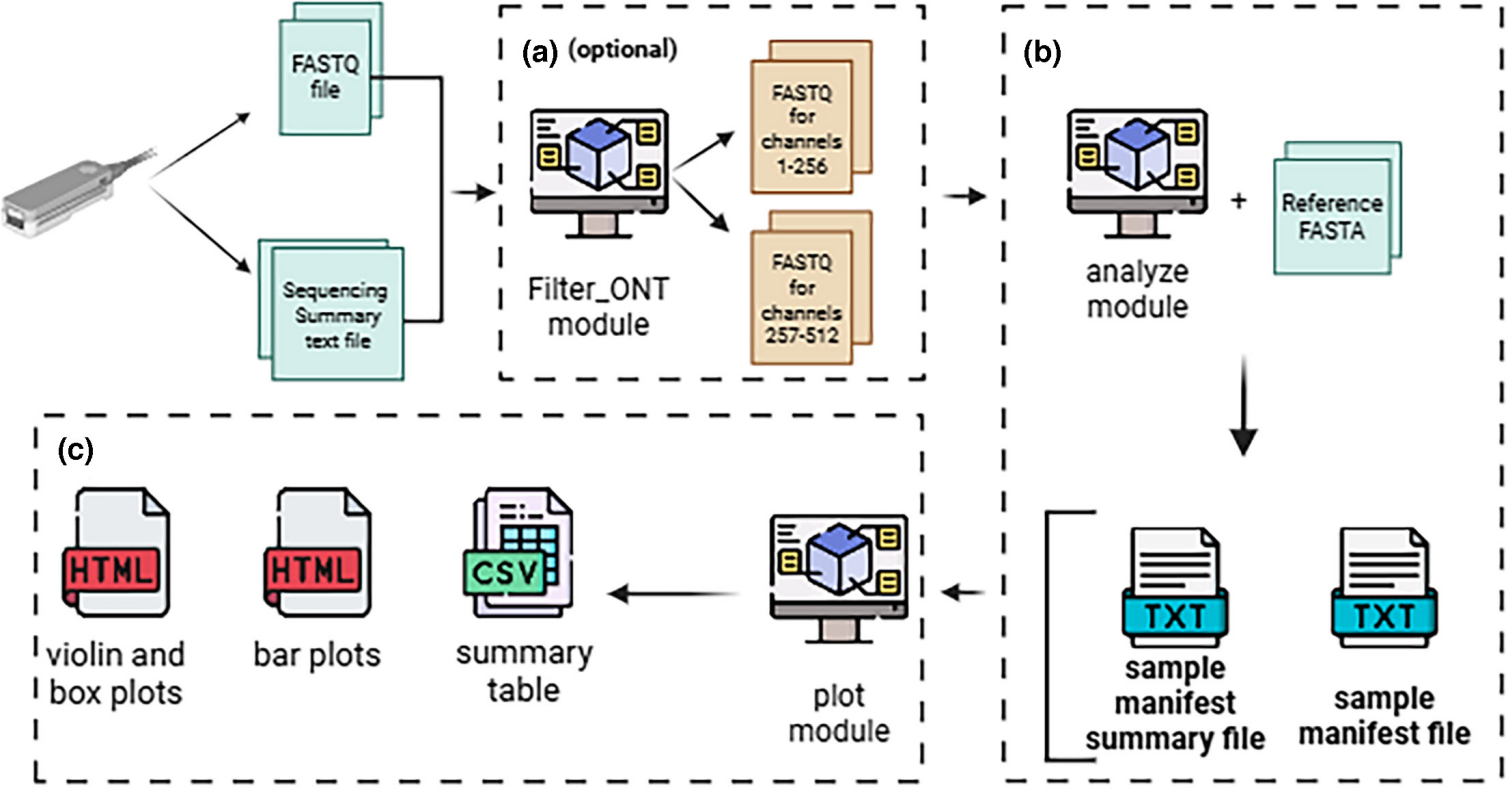
Overview of the Sequenoscope pipeline. (a) The *filter_ONT* module (optional). The FASTQ file and sequencing summary file generated by ONT (e.g. via Guppy) can be used to extract read subsets based on user-defined criteria – such as channel ranges, Q scores or length – thereby creating new sets of FASTQ files for downstream analysis. (**b**) The *analyze* module. The FASTQ file (raw or filtered), reference FASTA (which can include multiple reference sequences representative of each taxa to be found) and an optional sequencing summary file generated by ONT are supplied to the *analyze* module. This module generates key analytical outputs (e.g. mapping files, MASH outputs, filtered FASTQ file and read lists), culminating in two final text files: the *sample manifest* (per-read data) and the *sample manifest summary* (aggregated run statistics such as coverage depth and read quality). (**c**) The *plot* module. The *sample manifest* and *sample manifest summary* files serve as inputs for generating interactive plots (e.g. violin/box plots and bar charts) and a *summary table* in comma-separated values (CSV) format. These visualizations highlight read quality, taxon mean coverage depth, pore decisions (if AS is used) and other sequencing performance metrics in an accessible format (*created with BioRender.com*).

Sequenoscope comprises three main modules that streamline data processing for ONT sequencing runs (Fig. 1). The optional *filter_ONT* module (Fig. 1a) subsets reads based on user‐defined criteria (e.g. channel range, read length and Q score) using the ONT *sequencing summary* file. The *analyze* module (Fig. 1b) aligns reads to the user-provided reference(s) and produces outputs which capture run metrics such as taxon mean coverage depth, per cent taxon covered at default threshold of 1X and mean read length. Finally, the *plot* module (Fig. 1c) uses the *manifest* files generated by the *analyze* module to produce comparative visualizations (e.g. bar and violin plots), enabling side‐by‐side evaluation of control and AS conditions.

### The *filter_ONT* module

The *filter_ONT* module uses the ONT sequencing summary file (generated during basecalling by Guppy or Dorado using POD5 files created by MinKNOW) to filter reads according to specified criteria for parameters such as read sequencing decision (e.g. unblocked, no decision and stop receiving), channel range, read length, Q score and start or duration time (Fig. 1a). Using user-defined filtering criteria, the ONT sequencing summary file along with the raw basecalled FASTQ file, *filter_ONT* produces a curated list of matching reads. **Seqtk (v0.5.0)** [15] uses this curated list of reads to generate new FASTQ files. This module is especially helpful for AS runs where different channel groups operate under separate conditions (e.g. depletion vs. control), allowing users to segregate reads and directly compare distinct experimental conditions on the same flow cell.

### The *analyze* module

The *analyze* module is the core component of the pipeline, producing two key text file outputs – the *sample manifest* and the *sample manifest summary –* capturing essential sequencing statistics (Fig. 1b). Inputs include a FASTQ file, a reference FASTA for read mapping and optionally an ONT sequencing summary file. By providing the ONT sequencing summary file, the outputs will contain pore‐level details (e.g. channel usage or AS decisions). The *sample manifest* files from the *analyze* module for test and control conditions are the inputs for the *plot* module.

#### Filtering and mapping of raw reads

First, the *analyze* module validates FASTQ file integrity and detects corrupted reads by checking format indicators (‘@’ on line one, ‘+’ on line three) and ensuring the sequence length of each read matches its Q score length. Invalid files halt and exit the process. Verified reads are filtered via **fastp (v0.23.2)** [16] (default settings, with deduplication enabled using -D). We selected **fastp** to prioritize user accessibility and broad compatibility, as it is a widely tested tool that provides sufficient performance for basic read quality filtering and trimming. However, we plan to incorporate **fastplong** support in future tool releases, allowing users to choose the best optimized processing tool for both long-read and short-read data types. Filtered reads are mapped to the reference FASTA with **minimap2 (v2.24)** [17] (using the -ax map-ont preset optimized for ONT single-end reads or the -ax sr preset for pair-end Illumina reads), generating a SAM file. The *k*-mer size is explicitly fixed at 15 (via the **minimap2** -k 15) as this is the community-established optimal *k*-mer size for noisy ONT reads [17], which is optimal in our primary single-end long-read use case. **SAMtools (v1.18)** [18] converts this SAM file into both BAM format and a FASTQ file of successfully mapped reads. We note that Illumina short-read compatibility is included mainly to allow the tool to handle mixed or legacy datasets; however, full cross-platform benchmarking is beyond the scope of this study.

#### Distance calculations

To determine metrics such as estimated genome size and coverage depth, the pipeline’s *analyze* module applies **MASH v2.3** [19] with a k‐mer size (-k) 27 and a default sketch size (-s) of 1,000, automatically integrating its output. While MASH provides reference-free estimates of the total community genome size directly from the raw reads, the taxon-specific read mapping metrics generated by the *analyze* module (e.g. mean coverage depth, per cent breadth of coverage and mean mapped read length) are also recorded in the *sample manifest summary* file.

#### *Sample manifest* generation

Within the *analyze* module, two output files capture the results. The *sample manifest* provides per‐read details – the alignment status of each read (mapped or unmapped), read length, Q score and optional ONT‐specific data (e.g. channel numbers and AS decisions). By contrast, the *sample manifest summary* aggregates statistics at the taxon or reference level, providing a condensed representation of the *sample manifest* file. Using **SAMtools v1.18** (specifically the *idxstats* subcommand) and **pysam v0.21.0**, the pipeline computes total mapped reads, taxon mean coverage depth and mean read length, while **MASH v2.3** estimates a reference-free, community-wide total genome size (*est_genome_size*) and average sequencing depth (*est_coverage*). These two MASH-derived metrics reflect overall sequencing completeness across the entire metagenomic community, independent of reference sequences, and are particularly useful for poorly characterized complex microbial samples, where users may only provide references for specific targets or where only a fraction of reads may map to known references. Relevant metrics are also integrated from **fastp v0.23.2** (e.g. read counts). Together, these two outputs supply both fine‐grained and high‐level insights into sequencing performance.

### The *plot* module

The *plot* module generates interactive visual comparisons of two FASTQ files, e.g. a test condition (e.g. AS) and a control (e.g. regular sequencing) (Fig. 1c). The *plot* module uses the Python Plotly graphing library **v5.17.0** [14] to produce the plots. The *analyze* output directory for each experimental condition (i.e. *sample manifest* file and *sample manifest summary* file) is provided as input. An optional **–AS** parameter integrates AS details from the ONT sequencing summary file, if provided. By default, the *plot* module creates five interactive HTML plots: two violin plots (*read length and quality*) and three bar charts (*mean read length*, *mean coverage* and *per cent bases covered at 1X*). Note that the minimum coverage depth default threshold of 1X could be customized by the **--minimum_coverage** parameter in the *analyze* module. Further, a comma-separated values table, *summary table*, is generated. The *summary table* compares metrics between test and control FASTQ files, providing a quick overview of metrics. In addition, two *stacked bar charts* (independent and cumulative) are generated if **–AS** is specified; these charts illustrate real-time sequencing decisions (‘unblocked’, ‘no decision’ and ‘stop receiving’).

#### Stacked bar charts of sequencing decisions

There are two types of stacked bar charts of the sequencing decisions: independent (e.g. Fig. S3C-D, available in the online Supplementary Material) and cumulative (e.g. Fig. S3A-B). Each chart displays user-specified time interval bins on the x-axis, with two y-axes: percentage of sequencing decisions (left) and read count as a line graph (right). The *independent bar chart* depicts the distribution of sequencing decision percentages and the total read counts within discrete, non-overlapping time intervals. In contrast, the *cumulative bar chart* presents the accumulated decision distribution and total read counts, where data from each successive time bin builds upon the previous, offering a continuous cumulative representation of decisions over time. To produce these plots, sequencing decisions, as presented in the ‘end_reason’ column in the sequencing summary file, are utilized.

The ONT sequencing summary file logs per-read ‘end_reason’ values, indicating the pore-level reason why each read terminated sequencing. Sequenoscope uses these ‘end_reason’ values to map reads to three main initial real-time AS outcomes [‘stop_receiving’ (accept), ‘unblock’ (reject) and ‘no_decision’ (undecided)], consistent with definitions provided in prior studies [20]. Because the sequencing summary file does not explicitly store ReadUntil API per-read decisions, this mapping is approximate:

1. **‘Stop_receiving’ (accepted)**: This decision is assigned when the ‘end decision’ column for a read is set to ‘signal_positive,’ indicating that the sequencing of the DNA strand has been completed successfully (‘stop_receiving’) without being unblocked. As described by Vries *et al*. [20], the ‘signal positive’ category includes both reads that were explicitly accepted (‘stop_receiving’) and reads for which no decision was made (‘no_decision’) but nonetheless were sequenced to full length. The ‘stop_receiving’ status means that the pore continues sequencing until it detects the end of the read, at which point it stops receiving data because the sequencing process is complete.
2. **‘Unblocked’ (rejected)**: This decision is associated with the ‘data_service_unblock_mux_change’ value, which occurs when the pore rejects the current read and prepares to allow entry of a new strand. This status differs from the ‘unblock_mux_change’ in that ‘data_service_unblock_mux_change’ specifically refers to the action of unblocking the pore after receiving a decision to reject a read, allowing the pore to reset and eject the strand by reversing the pore voltage and getting ready to accept a new strand.
3. **‘No_decision’ (not fully accepted or rejected)**: This approximate status represents reads for which ReadUntil API did not issue an explicit read accept (‘stop_receiving’) or reject (‘unblock’) decision during the early read evaluation window. Sequenoscope infers this read classification category indirectly from the sequencing summary file ‘signal_negative’ and ‘unblock_mux_change’ values. A ‘signal_negative’ indicates that the current detected during sequencing was insufficient to classify the event as a proper sequencing read, often because the current drop was less than expected for a valid sequencing event. In such cases, the pore does not make a definitive sequencing decision, and the strand remains in the pore without triggering further sequencing activity. These ‘end_reason’ values (mapping to ‘signal_negative’ or ‘unblock_mux_change’) indicate that the pore did not perform an active read accept or reject action, resulting in reads that were neither explicitly enriched nor depleted by the AS process.

It is important to note that the ‘end_reason’ values in the *sequencing summary* file are not fully equivalent to the true AS initial read decision states generated by the ReadUntil API but instead reflect downstream pore-level termination states. In many ONT runs, the optional adaptive_sampling_summary log file is not always produced, and therefore, Sequenoscope can only rely on ‘end_reason’ final state read annotations as a practical retrospective AS run proxy. For users who have access to the *AS summary* log file and want more specificity, future versions of Sequenoscope will natively support both modes of operation: (i) approximate read classification based on the *sequencing summary* file (used in this study) and (ii) direct use of AS decision logs (i.e. *AS summary* file) when available.

### Experimental design

#### Library preparation and sequencing

Triplicate reactions of 50 ng of ZymoBIOMICS Microbial Community DNA Standard II (Log Distribution) (Cat# D6311) and ZymoBIOMICS Microbial Community DNA Standard (Even Distribution) (Cat# D6306) were prepared for sequencing with ONT Rapid Barcoding Kit 24 v14 (SQK-RBK114.24) according to the manufacturer’s directions and assigned barcodes 1–3 and 4–6, respectively. The prepared library was loaded on an ONT MinION R10.4.1 flow cell according to the manufacturer’s best practice, and sequencing was performed using MinKNOW GUI v23.04.6. Live high-accuracy basecalling was performed with Guppy v6.5.7 at 400 bp sequencing speed. The minimum accepted Q score was set to 9 in MinKNOW. The flow cell channels were split, with 256 channels sequencing all strands (control) and the other 256 channels performing AS, depleting for *Listeria monocytogenes* (test). The AS settings were configured with a minimum decision read length of 200 bp with a default decision window of 1 s corresponding to ~400 bp [8]. The *L. monocytogenes* reference genome (NCBI accession CP117973), obtained from the ZymoBIOMICS standards library, was supplied to MinKNOW in FASTA format. *L. monocytogenes* comprises 89.1% of the log distributed and 12% of the evenly distributed community standard. Sequencing data was collected for 39 h. Following sequencing, for each barcode, FASTQ files were concatenated into a single FASTQ file utilizing the cat command in a Linux environment.

#### Pipeline application to long-read ONT AS data

The three modules of Sequenoscope were run on all six barcodes (Table 1). The *filter_ONT* module was used to separate the reads from the two different experimental conditions, control (min_ch=257 and max_ch=512) and test (min_ch=0 and max_ch=256). The output was a FASTQ file for each experimental condition (i.e. two FASTQ files per barcode). The *analyze* module was run independently for each FASTQ file generated with the *filter_ONT* module; a reference FASTA containing the eight bacterial chromosomes from the ZymoBIOMICS standards was also provided (https://zymo-files.s3.amazonaws.com/BioPool/ZymoBIOMICS.STD.refseq.v2.zip). The large eukaryotic genomes of *Cryptococcus neoformans* and *Saccharomyces cerevisiae* were excluded from the reference FASTA. This exclusion was intentional given that our primary research focus is on bacterial taxa, and therefore, we would only provide those reference genomes of interest to Sequenoscope and not reference genomes for all taxa present in the metagenomic sample. We consider the risk of spurious read mapping to be minimal, as within the ZymoBIOMICS EVEN and LOG mock community standards, the two excluded eukaryotic organisms are present at low relative abundance, ranging from a maximum of 2% (EVEN) down to 0.00089% (LOG) (Table 1). Nevertheless, we acknowledge that omitting specific reference taxa can theoretically lead to occasional off-target spurious mapping of reads from these excluded organisms to conserved regions of the included bacterial genomes potentially inflating taxa abundance estimates [21]. With the references selected, the *analyze* module was run twice for each barcode: once on the test/AS FASTQ file and once on the control FASTQ file to generate the two distinct data conditions required by the *plot* module for comparative visualization.

**Table 1.** Details on barcode, replicates and sample The barcode layout used in the study specifies the microbial ZymoBIOMICS mock community standard applied. Taxonomic composition and theoretical relative abundances are based on genomic DNA (gDNA) concentrations as defined by the manufacturer.

FASTQ file	Barcode no.	Sample	Taxon composition	Relative abundance (%)	Reference genome size (Mb)
Log 1 control	Barcode 1	ZymoBIOMICS Microbial Community Standard II (Log Taxa Distribution)	*L. monocytogenes* *Pseudomonas aeruginosa* *Bacillus subtilis* S. cerevisiae* *Escherichia coli* *Salmonella enterica* *Lactobacillus fermentum* *Enterococcus faecalis* C. neoformans* *Staphylococcus aureus*	89.1 8.9 0.89 0.89 0.089 0.089 0.0089 0.00089 0.00089 0.000089	2.99 6.79 4.05 12.84 4.88 4.76 1.91 2.85 29.18 2.73
Log 1 test					
Log 2 control	Barcode 2				
Log 2 test					
Log 3 control	Barcode 3				
Log 3 test					
Even 1 control	Barcode 4	ZymoBIOMICS Microbial Community Standard (Even Taxa Distribution)	*L. monocytogenes* *Pseudomonas aeruginosa* *Bacillus subtilis* S. cerevisiae* *Escherichia coli* *Salmonella enterica* *Lactobacillus fermentum* *Enterococcus faecalis* C. neoformans* *Staphylococcus aureus*	12 12 12 2 12 12 12 12 2 12	2.99 6.79 4.05 12.84 4.88 4.76 1.91 2.85 29.18 2.73
Even 1 test					
Even 2 control	Barcode 5				
Even 2 test					
Even 3 control	Barcode 6				
Even 3 test					

* Eukaryotic taxa (*S. cerevisiae* and *C. neoformans*) were excluded from the AS reference FASTA and therefore were not targeted during test and control AS runs.

Finally, the *plot* module, invoked with **–AS** parameter, compared the test and control outputs from the *analyze* module. The *plot* module was provided with the ONT sequencing summary file as well as the *sample manifest* file and *sample manifest summary* file for both the test and control/AS FASTQ files. The **–test_dir** parameter pointed to the AS directory and **–control_dir** to the control directory, enabling side‐by‐side visualization of sequencing performance.

#### Pipeline application to short-read Illumina data

To demonstrate Sequenoscope’s cross-platform compatibility, we performed a proof-of-principle validation using publicly available Illumina HiSeq 2500 shotgun sequencing data from the ZymoBIOMICS mock community standards [22]. Specifically, we downloaded the log-distributed (accession: ERR2935805) and evenly distributed (accession: ERR2984773) short-read datasets and successfully processed them through the Sequenoscope *analyze* and *plot* modules, generating all expected outputs including sample manifests, summary tables and interactive plot visualizations.

## Results and discussion

In this use-case study, Sequenoscope was tested with an ONT AS dataset consisting of two samples (log and even ZymoBIOMICS Microbial Community DNA Standards) each sequenced in triplicate (six barcodes) under control and test (AS depletion of *L. monocytogenes*) conditions giving a total of 12 FASTQ files (Table 1). While all six barcodes were tested with Sequenoscope, we are only presenting representative examples of Sequenoscope outputs from barcode 1 (Log 1 control and test) and barcode 5 (Even 1 control and test) within the main text. Similar trends were observed for the additional technical replicates. Equivalent outputs for all remaining replicates are available in the online Microbiology Society Figshare Supplemental Materials repository [Tables S1–S30 (in Excel format) and Figs S1–S18 (in PDF format)]. Tables S1–S30 representing outputs from barcodes 1–6 include the following: S1–S12, short *sample manifest summaries*; S13–S24, complete *sample manifests* both generated as a primary output of the *analyze* module; and S25–S30, *summary tables* generated by the *plot* module.

**Table 2.** *Test manifest summary* for Log 1 test replicate (barcode 1) An example of the adapted *sample manifest summary* file, an output from the *analyze* module for the Log 1 replicate under test conditions. Asterisk (*) denotes unclassified reads which are removed during the mapping phase. The term ‘taxon’ and related metrics are utilized to describe any included reference whether it corresponds to a species, strain, plasmid or other genomic entity, and in this dataset, each taxon corresponds to a species. Taxon_id was abbreviated compared to the original file. Taxon_mean_coverage (taxon mean coverage depth) and taxon_%_covered_bases_1X (per cent taxon coverage breadth) were rounded to two significant digits. Taxon_mean_read_length was rounded to the nearest whole number.

taxon_id	taxon_ length	taxon_mean_coverage	taxon_covered _bases_1X	taxon_%_ covered_ bases_1X	total_taxon_ ref_mapped_bases	taxon_mean_read_length
*B. subtilis*	4,045,677	1.0	2,448,134	61	4,042,750	1,830
*E. faecalis*	2,845,392	0.33	60,378	2.1	927,924	286
*E. coli*	4,765,434	0.12	482,894	10	552,035	2,841
*L. fermentum*	1,905,333	0.011	17,878	0.94	20,264	1,027
*L. monocytogenes*	2,992,342	33	2,992,342	100	99,132,820	514
*P. aeruginosa*	6,792,330	4.4	6,700,968	99	30,146,240	2,145
*S. enterica*	4,759,746	0.083	371,007	7.8	392,725	2,063
*S. aureus*	2,718,780	0.0019	5,255	0.19	5,255	1,432
*	0	0	0	0	0	0

The following sample-wide metrics were applied to all taxa (rows) of the Log 1 test replicate (barcode 1): MASH estimated community-wide total genome size (*est_genome_size*) = 7,507,750 bp and average sequencing depth (*est_coverage_size*) = 11.76X; total raw bases after basecalling (*total_bases*) = 1.37E+08 bp; and total bases (*total_fastp_bases*) = 1.33E+08 and mean read length (*mean_read_length*) = 685 bp after **fastp** filtering.

**Table 3.** Subset of the *sample manifest* file for the Log 1 test replicate (barcode 1) A subset of seven reads from the *sample manifest* file output of the *analyze* module for the Log 1 replicate under test conditions. Raw values for read_qscore, start_time and end_time have been rounded to the nearest 100th for clarity. is_mapped refers to whether or not a read is mapped to any sequence in the multi-sequence FASTA reference file provided by the user. If true, the contig is provided in the contig_id column. is_uniq refers to whether or not a read is unique throughout the sample manifest file. In ONT sequencing, a read may be processed multiple times if the decision is labelled as ‘signal_negative’ or ‘no_decision’ before a final decision is made on whether to allow the read to continue sequencing.

sample_id	read_id	read_len	read_qscore	Channel	start_time	end_time	Decision	fastp_status	is_mapped	is_uniq	contig_id
Test	a11b90e2-1044-4751-a46a-330e1165c950	825	14.04	232	48.34	50.62	Signal_positive	True	True	True	Listeria_monocytogenes
Test	09186102-12e8-42e6-9232-d84df36b23a4	272	11.29	88	50.16	51.33	Signal_positive	True	True	True	Listeria_monocytogenes
Test	7c90ddbd-7e30-4f6f-bcd6-27574e8fc0df	1342	9.13	211	108.37	111.55	Signal_positive	False	False	True	
Test	a373beb8-0827-4390-9e6b-b74c69b62bdb	1404	14.94	255	86.13	91.52	Signal_positive	True	True	True	Pseudomonas_aeruginosa
Test	a537b181-2f26-4c7c-b6bd-361dda8d5a82	2039	15.16	19	185.07	190.84	Signal_positive	True	True	True	Escherichia_coli
Test	73a9bfb2-1dd4-4936-8e63-9b2b6a0e6d9f	383	9.34	247	204.24	205.44	Signal_positive	False	False	True	

**Table 4.** *Summary table* for Log 1 replicate An example of the *summary table*, an output from the *plot* module for the Log 1 replicate. The term ‘taxon’ and related metrics are utilized to describe any supplied reference in FASTA format whether it corresponds to a species, strain, plasmid or other genomic entity, and in this dataset, each taxon corresponds to a species. Taxon_id was abbreviated compared to the original file. Taxon_mean_coverage (taxon mean coverage depth) was rounded to two decimal places. Taxon_%_covered_bases_1X (per cent taxon coverage breadth), taxon_covered_bases_1X (taxon coverage breadth), taxon_mean_read_length and total_taxon_ref_mapped_bases were rounded to the nearest whole number.

taxon_id	Parameter	test_Value	control_Value
*B. subtilis*	taxon_%_covered_bases_1X	61	50
*B. subtilis*	taxon_covered_bases_1X	2,448,134	2,018,419
*B. subtilis*	taxon_mean_coverage	1.00	0.73
*B. subtilis*	taxon_mean_read_length	1,830	1,779
*B. subtilis*	total_taxon_ref_mapped_bases	4,042,750	2,940,354
*E. faecalis*	taxon_%_covered_bases_1X	2	2
*E. faecalis*	taxon_covered_bases_1X	60,378	49,546
*E. faecalis*	taxon_mean_coverage	0.33	0.84
*E. faecalis*	taxon_mean_read_length	286	887
*E. faecalis*	total_taxon_ref_mapped_bases	927,924	2,395,066
*E. coli*	taxon_%_covered_bases_1X	10	8
*E. coli*	taxon_covered_bases_1X	482,894	388,050
*E. coli*	taxon_mean_coverage	0.12	0.09
*E. coli*	taxon_mean_read_length	2,841	2,139
*E. coli*	total_taxon_ref_mapped_bases	552,035	415,032
*L. fermentum*	taxon_%_covered_bases_1X	1	1
*L. fermentum*	taxon_covered_bases_1X	17,878	9,986
*L. fermentum*	taxon_mean_coverage	0.01	0.01
*L. fermentum*	taxon_mean_read_length	1,027	990
*L. fermentum*	total_taxon_ref_mapped_bases	20,264	9,986
*L. monocytogenes*	taxon_%_covered_bases_1X	100	100
*L. monocytogenes*	taxon_covered_bases_1X	2,992,342	2,992,342
*L. monocytogenes*	taxon_mean_coverage	33.13	89.47
*L. monocytogenes*	taxon_mean_read_length	514	1,842
*L. monocytogenes*	total_taxon_ref_mapped_bases	99,132,820	267,721,800
*P. aeruginosa*	taxon_%_covered_bases_1X	99	96
*P. aeruginosa*	taxon_covered_bases_1X	6,700,968	6,528,942
*P. aeruginosa*	taxon_mean_coverage	4.44	3.39
*P. aeruginosa*	taxon_mean_read_length	2,145	2,135
*P. aeruginosa*	total_taxon_ref_mapped_bases	30,146,240	23,014,055
*S. enterica*	taxon_%_covered_bases_1X	8	7
*S. enterica*	taxon_covered_bases_1X	371,007	356,391
*S. enterica*	taxon_mean_coverage	0.08	0.08
*S. enterica*	taxon_mean_read_length	2,063	2,450
*S. enterica*	total_taxon_ref_mapped_bases	392,725	399,086
*S. aureus*	taxon_%_covered_bases_1X	0	0
*S. aureus*	taxon_covered_bases_1X	5,255	4,463
*S. aureus*	taxon_mean_coverage	0.00	0.00
*S. aureus*	taxon_mean_read_length	1,432	1,363
*S. aureus*	total_taxon_ref_mapped_bases	5,255	4,463
* (unmapped)	taxon_%_covered_bases_1X	0	0
* (unmapped)	taxon_covered_bases_1X	0	0
* (unmapped)	taxon_mean_coverage	0.00	0.00
* (unmapped)	taxon_mean_read_length	0	0
* (unmapped)	total_taxon_ref_mapped_bases	0	0

The asterisk (*) in the *taxon_id* column denotes sequencing reads that did not align to any of the provided reference genomes (i.e. unmapped reads).

**Table 5.** Relative taxa abundance for Illumina-sequenced LOG and EVEN ZymoBIOMICS mock community standards Comparison of observed against expected relative per cent abundances for the LOG (accession: ERR2935805) and EVEN (accession: ERR2984773) ZymoBIOMICS microbial community standards. Relative abundance was calculated as the percentage of taxon-specific sequenced bases relative to the total bases sequenced within the community. The results demonstrate the pipeline’s support for short-read Illumina data, validating its ability to perform accurate taxonomic profiling of metagenomic samples.

Taxon ID	LOG standard		EVEN standard	
	Observed (%)	Expected (%)	Observed (%)	Expected (%)
*Bacillus subtilis*	0.85	0.89	12.35	12
*Enterococcus faecalis*	0.58	0.00089	12.27	12
*Escherichia coli*	0.0729	0.089	12.76	12
*Lactobacillus fermentum*	0.0089	0.0089	11.12	12
*L. monocytogenes*	87.95	89.1	11.92	12
*Pseudomonas aeruginosa*	10.45	8.9	16.03	12
*Salmonella enterica*	0.08	0.089	13.34	12
*Staphylococcus aureus*	0.0037	0.000089	10.21	12

To demonstrate Sequenoscope’s cross-platform compatibility, we performed a proof-of-principle validation using publicly available Illumina HiSeq 2500 short-read sequencing data. Specifically, log-distributed (LOG) and evenly distributed (EVEN) ZymoBIOMICS mock communities standards [22] were used to demonstrate the pipeline’s ability to process standard, non-AS data while supporting core comparative metrics and visualizations as detailed in Validation on short-read public Illumina data.

### The *filter_ONT* module

The *filter_ONT* module was used to split the reads for each barcode into test and control FASTQ files based on the flow cell channel. Different flow cell channels were set to run the test and control conditions on the same flow cell/barcode to minimize batch effects. Channels 1–256 were allocated for the test condition and channels 257–512 for the control. The channel‐based partitioning in the *filter_ONT *allows users to have separate FASTQ files for comparison of test and control conditions without having to run test and control conditions on different library preparations or flow cells minimizing variability. Although this module is limited to ONT data, it could be used on its own for users that want to split FASTQ files based on channel range, sequencing decision, read length, Q score and time (start or duration).

### The *analyze* module

For each FASTQ file generated with *filter_ONT*, the *analyze* module provided the following: a *sample manifest summary* (Table 2, example from Log 1 test) offering aggregated coverage depth metrics per taxa and a *sample manifest* (Table 3, limited example from Log 1 test) presenting read‐level details. The *sample manifest summary* and *sample manifest* (limited example) for Log 1 test are provided in Tables 2 and 3, respectively; an Excel version of the *sample manifest* and *sample manifest summary* files for all FASTQ files is provided in Tables S1–S24.

For Log 1 test, the *sample manifest summary* showed a mean coverage depth of 33.1X for *L. monocytogenes*, while the mean coverage depth for most other taxa was less than 1X (Table 2). The percentage of the genome covered at 1X (i.e. coverage breadth) was mostly <10% for the majority of the mock community taxa; however, *Bacillus subtilis*, *Pseudomonas aeruginosa* and *L. monocytogenes* reached 1X coverage breadth of 61%, 98% and 100% of their genomes, respectively (Table 2). Average read length varies by taxa, but most taxa had an average >1,000 bp. However, *L. monocytogenes* and *Enterococcus faecalis* had average read lengths of 514 and 286, respectively (Table 2). A lower average read length was expected for *L. monocytogenes* as the test channels on the flow cells under AS conditions were actively depleting this species, but the shorter read length observed for *E. faecalis* was unexpected. Although *E. faecalis* shares 1.96% genome-wide homology with *L. monocytogenes*, homology alone cannot account for this observed pattern. Additional factors may have contributed, including taxon misassignment of reads due to the short decision window (~400 bp) that originate from conserved interspecies genomic regions shared across Gram-positive taxa or stochastic pore-availability effects, where rapid rejection of abundant *L. monocytogenes* reads under AS conditions increases opportunities for short fragments from rare taxa to enter sequencing pores. We also note that *E. faecalis* exhibits the shortest average read length under both test (285 bp) and control (998 bp) conditions (Tables 2, S2 and S3), suggesting that its DNA may have been partially fragmented prior to library preparation or more prone to shearing. Such short-read fragments can be difficult to classify correctly because their early sequence signal often lacks sufficient taxa-specific information. Under these conditions, the ReadUntil API may erroneously interpret the initial signal (typically corresponding to the first 200–400 bp of sequencing) as the depletion target (*L. monocytogenes*) and issue a ‘stop receiving’ decision even when the sequenced read fragment originates from *E. faecalis*. Likewise, **minimap2** may subsequently misassign these short reads because of their limited length having too few unique *k*-mers to unambiguously distinguish closely related Gram-positive taxa. Observations like these highlight opportunities for users to refine parameters to further improve performance in AS workflows. Practical mitigation strategies could include the following: refining the reference database by masking conserved inter-taxa genomic regions; clustering highly similar reference genomes and using a consensus or centroid sequence to represent each cluster, thereby reducing ambiguity; and exploring alternative alignment parameters (e.g. larger minimap2 *k*-mer sizes or modified decision thresholds) to enhance species-level discrimination. Incorporating such reference-optimization approaches may help reduce unintended depletion/enrichment of closely related taxa as a streamlined reference database enhances computational efficiency, leading to faster AS per-read decisions and a reduction in ‘no-decision’ reads [8,23]. Together, these observations illustrate how the *analyze* module can be used to flag confounding hidden issues with the experimental AS conditions that may not be evident using other analysis methods.

Table 3 presents a *sample manifest* excerpt for Log 1 test, which highlights per‐read fields such as read length, Q score, mapping status and AS decisions. This level of granularity allows users to identify anomalies or quality issues at the read level – for instance, verifying whether specific reads mapped to the targeted reference or were unblocked.

Together, the *sample manifest summary* and *sample manifest* files capture details about a single FASTQ file such as high‐level per-taxon trends (e.g. mean coverage depth, per cent taxon coverage and average read length) and fine details per‐read outcomes. In addition, the module accepts short-read data including Illumina for users wishing to test two experimental laboratory conditions that are sequenced on a different platform. The *analyze* module allows users to better assess not only AS FASTQ files but any FASTQ files in comparison to a reference FASTA.

### The *plot* module

The *plot* module produces comparisons, both graphical and numerical, between two FASTQ files, test and control, using the outputs from the *analyze* module. Below, we describe its three primary outputs: *taxon mean coverage depth bar charts* (Figs2  3), *summary table* (Table 4) and *independent decision bar charts* (Figs4  5). A PDF version of the graphical and an Excel version of the numerical *plot* module outputs for the six FASTQ file comparisons are provided in Figs S1–S18 and Tables S25–S30, respectively. This includes plots for *mean read length*, *per cent taxon coverage*, *overall read length* and *overall Q score*. For comparisons including AS as the test condition, these outputs show how mean coverage depth and real‐time read decisions are impacted by the test conditions.

**Fig. 2.**
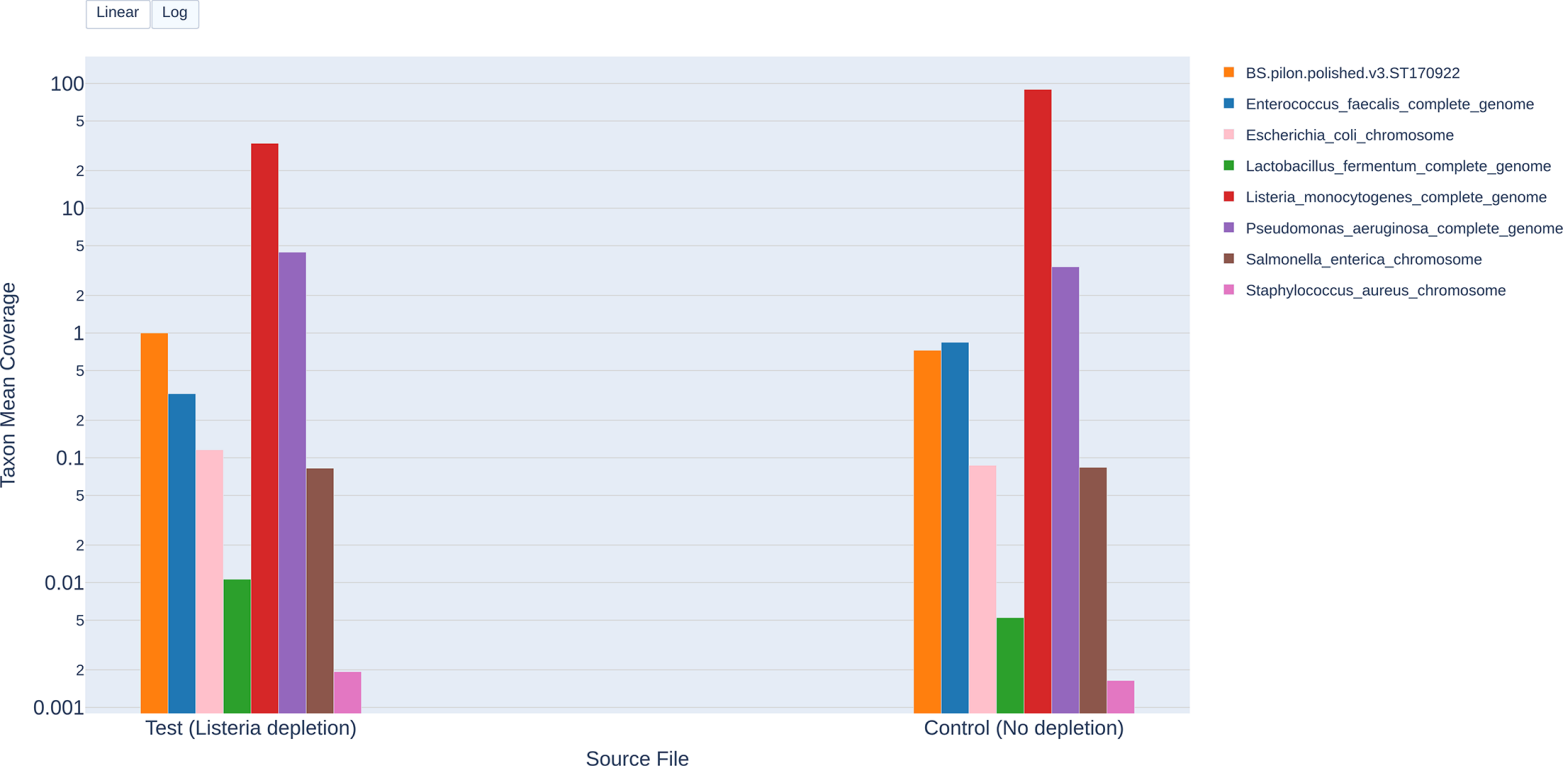
Comparative *mean coverage depth plot* for the ZymoBIOMICS Log 1 replicate using AS depletion. This bar chart shows per‐taxon mean coverage depth for the test FASTQ file (reads from channels 1–256, AS depletion of *L. monocytogenes*) versus the control FASTQ file (reads from channels 257–512, no depletion). A toggle on the plot allows switching between linear and logarithmic scales on the y‐axis, revealing differences in coverage depth between high‐ and low‐abundance taxa. The y-axis is presented on a logarithmic scale to clearly visualize the coverage depth across both high- and low-abundance taxa.

**Fig. 3.**
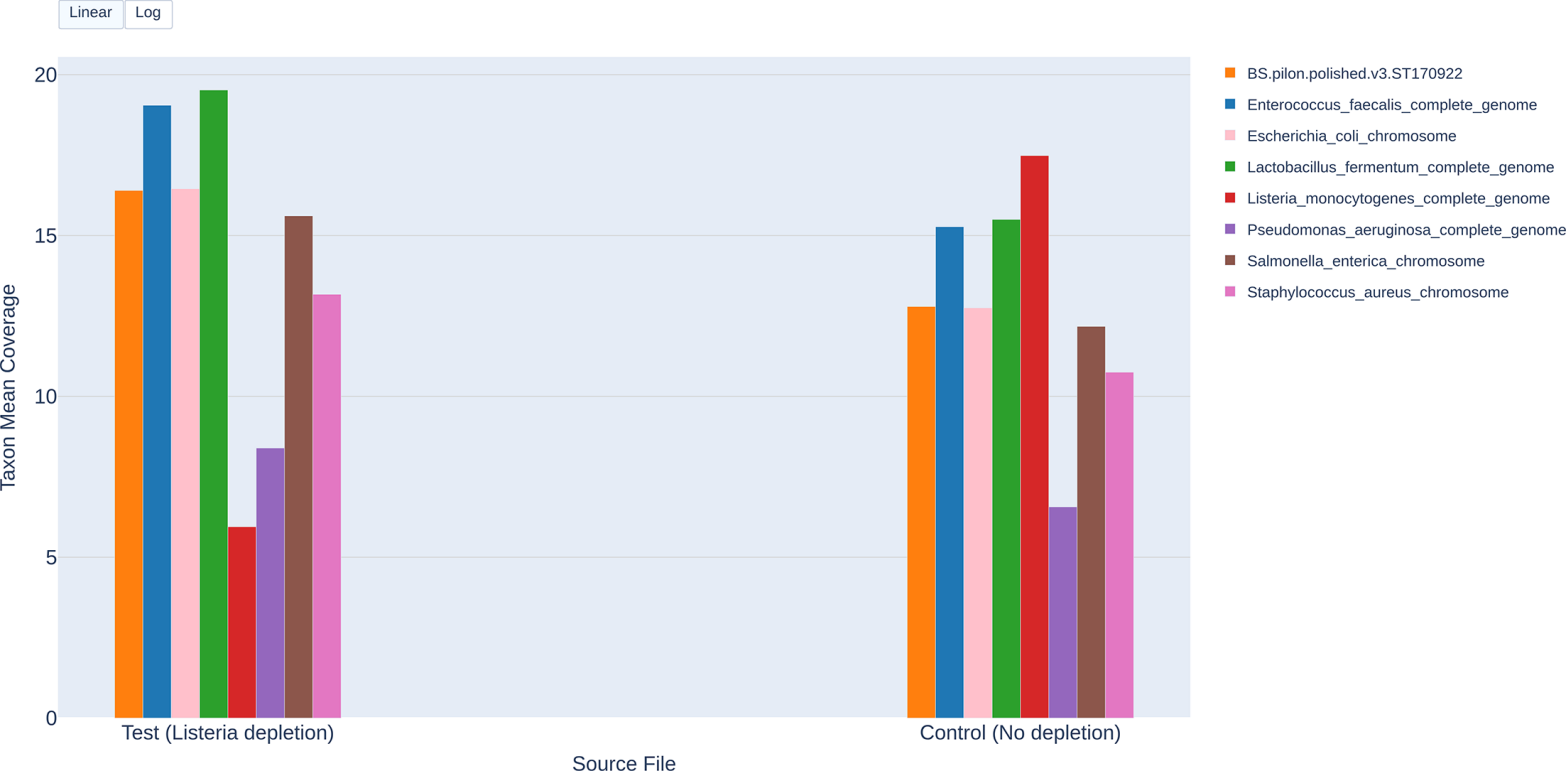
Comparative *mean coverage depth plot* for the ZymoBIOMICS Even 2 replicate using AS depletion. This bar chart shows per‐taxon mean coverage depth for the test FASTQ file (reads from channels 1–256, AS depletion of *L. monocytogenes*) versus the control FASTQ file (reads from channels 257–512, no depletion). The y‐axis is presented on a linear scale.

**Fig. 4.**
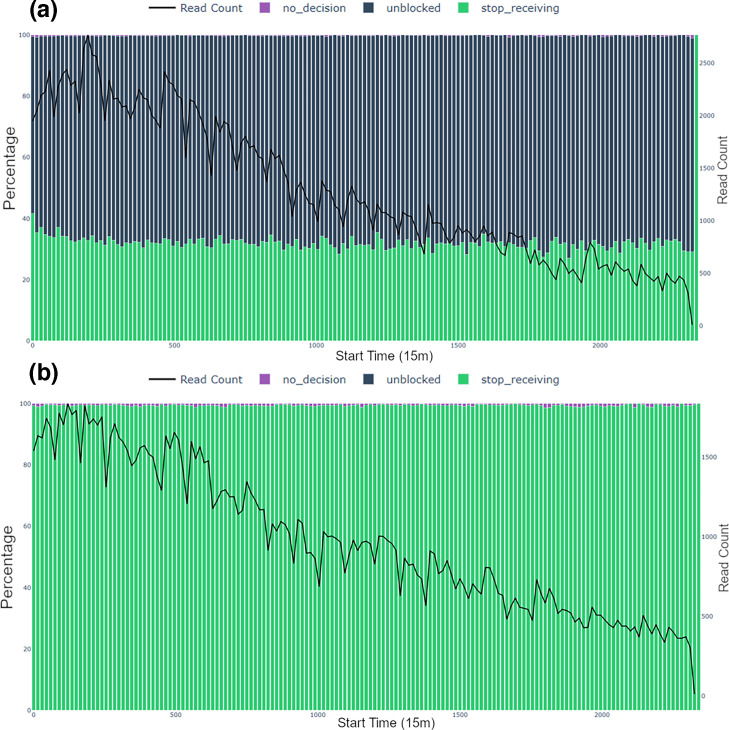
*Independent decision bar charts* for the Log 1 replicate using 15-min time bins. Panels (a) test/AS (*L. monocytogenes* depletion) and (b) control (no depletion) summarize ONT read decisions by time bin, with the black line showing read count in each interval. Bar colours represent the percentage of reads for each status: ‘unblocked’ (blue), ‘no_decision’ (purple) and ‘stop_receiving’ (green). See Methods for more details on these terms.

**Fig. 5.**
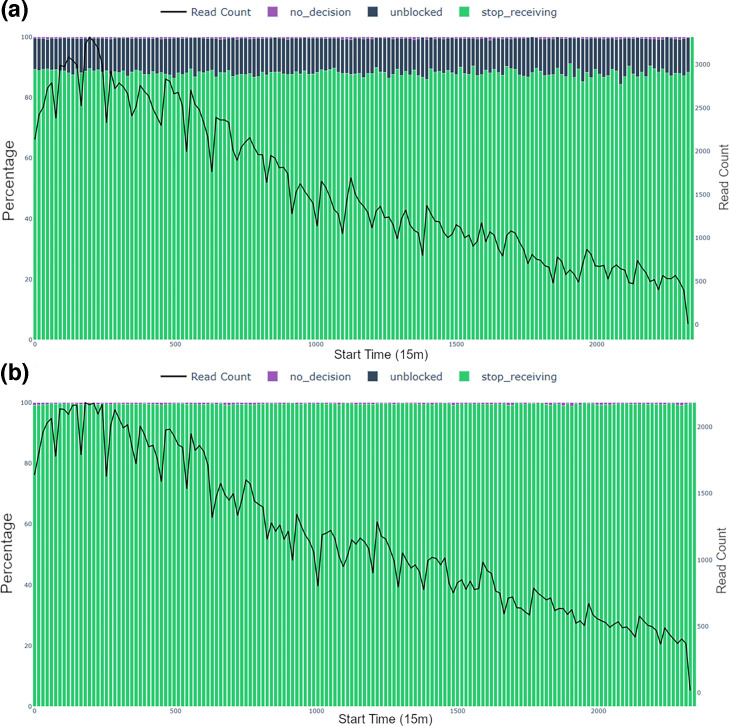
*Independent decision bar charts* for the Even 2 replicate using 15-min time bins. Panels (a) test/AS (*L. monocytogenes* depletion) and (b) control (no depletion) summarize ONT read decisions by time bin, with the black line showing read count in each interval. Bar colours represent the percentage of reads for each status: ‘unblocked’ (blue), ‘no_decision’ (purple) and ‘stop_receiving’ (green). See Methods for more details on these terms.

#### Mean coverage depth bar charts

Fig 2 (Log 1 replicate) and Fig. 3 (Even 2 replicate) present side‐by‐side comparisons of the mean coverage depth for each taxa under AS (test) versus no depletion (control). In Log 1 replicate (Fig. 2), the log toggle for the plot was used to enable better visualization of low-abundance taxa. Further, there was a noticeable decrease in the *L. monocytogenes* coverage depth between the control and test, and a small, but noticeable increase in the coverage depth of most of the other taxa (Fig. 2). In Even 1 replicate (Fig. 3), the linear scale was used. *L. monocytogenes* coverage depth dropped between the control and test, and a slight increase was seen in the coverage depth of other taxa (Fig. 3). In both sample types (log and even), the drop in coverage depth of *L. monocytogenes* points to successful species depletion via AS. For most of the non‐depletion targeted taxa, coverage depth slightly increased in the test over the control, underscoring that AS was specifically depleting *L. monocytogenes*. The log‐scale toggle (used in Fig. 2) was useful in visualizing coverage depth differences when comparing taxa with large differences in their abundance, while linear scaling worked well for a community with more equal representation of taxa. Our use case study showed targeted depletion and highlighted the ability of the *plot* module to provide quick visual evidence of the impact of AS on taxon coverage depth.

#### Summary table

The *plot* module *summary table* output compares metrics such as taxon mean coverage depth, total mapped bases and per cent of taxon bases covered between test and control at the default customizable threshold of 1X (Table 4). While the *mean coverage depth bar chart* visually shows a comparative visual overview, the *summary table* shows the actual magnitude of the change. The *L. monocytogenes* coverage depth decreased from 89.46X (control) to 33.1X (test) in the Log 1 replicate and from 17.8X (control) to 5.94X (test) (Tables 4 and S29) in the Even 2 replicate, again demonstrating successful depletion of the target taxon, *L. monocytogenes*. Similar to the *sample manifest summary*, the *summary table* can highlight potential issues such as those observed for *E. faecalis*. The *sample manifest summary* showed that the mean read length for *E. faecalis* was low compared to the other taxa; the *summary table* showed how the mean read length differs between the test (286 bp) and control (887 bp, Table 4) conditions, further suggesting depletion of *E. faecalis* in the test. This side‐by‐side format helps pinpoint which taxa are most affected by AS, by how much and in which direction.

#### Independent decision bar chart

The decision dynamics during the sequencing run for two FASTQ files are illustrated in Fig. 4 (Log 1, log-distributed standard technical replicate 1) and Fig. 5 (Even 2, evenly distributed standard technical replicate 2). For Log 1 replicate (Fig. 4), ‘unblocked’ (sequencing stopped, read ejected from pore) decisions accounted for 57–72% of all sequencing events in the test FASTQ file (Fig. 4a) in contrast to no ‘unblocked’ outcomes across the same run in the control FASTQ file (Fig. 4b). This is indicative of a sequencing workflow where active rejection was occurring in the test run and no AS was taking place in the control run. Similarly, for Even 2 replicate (Fig. 5), the decision bar charts demonstrated the rate of sequence rejection of *L. monocytogenes* between the two experimental conditions of test/AS (Fig. 5a) and control/standard sequencing (Fig. 5b). The effectiveness of the depletion strategy was demonstrated in this replicate as ‘unblocked’ decisions in a test dataset accounted for ~10–14% of all sequencing decisions (Fig. 5a). This plot highlights how AS influences decisions, particularly the prevalence of ‘unblocked’ actions in the test datasets (Figs 4a and 5a). Further, the decision bar charts can be compared between two test conditions, e.g. between Log 1 (Fig. 4) and Even 2 dataset (Fig. 5). The percentage ‘unblocked’ was much higher for Log 1 than Even 2 dataset. This was expected given that *L. monocytogenes* was present in 89.1% in the log sample and only 12% in the even sample. However, in other circumstances, this plot can be used to alert to issues with AS runs where more or less rejection is expected.

### Comparison with existing tools

The three-module Sequenoscope architecture provides unique, post-sequencing evaluation advantages not readily available in real-time AS instrument control software (e.g. ReadFish, ReadBouncer and MinKNOW) or general-purpose analysis platforms (e.g. EPI2ME). Specifically, it offers the following: (i) structured, per-taxon comparative metrics between test and control conditions, (ii) MASH *k*-mer-based reference-free QC metrics to assess total community complexity and (iii) comprehensive visualization of read decisions, which can be approximated from the sequencing summary’s end_reason values for increased flexibility when the AS log file is missing. These capabilities fill both analytical and visualization gaps, as existing tools typically focus on run control, enrichment execution or general read-mapping summaries rather than structured, experiment-level AS performance evaluation and validation.

### Validation on short-read public Illumina data

The *analyze* module successfully mapped the paired-end short-reads from [22] to the eight bacterial reference genomes, providing inputs for the *plot* module. To leverage the comparative functionality of the *plot* module, the ZymoBIOMICS mock community dataset with log-distributed taxa (LOG) was assigned as the test condition and the dataset with the evenly distributed community taxa (EVEN) as the control condition. In this configuration, the *plot* module successfully generated the mean coverage, per cent bases covered at 1X and mean read length bar charts which showed all eight expected bacterial taxa. These results accurately captured the expected abundance of taxa levels, matching the expected taxa distributions of LOG and EVEN mock community standards (see Tables 1 and 5). For the EVEN community standard, the estimated relative abundance, calculated as the proportion of taxon-specific mapped bases relative to all mapped bases, was balanced across all eight bacteria at ~12% each (ranging from 10.2 to 16%). Conversely, for the LOG community standard, the results correctly identified the highly skewed distribution where *L. monocytogenes* dominated at 87.95% followed by *P. aeruginosa* at 10.45% relative abundance, while the remaining taxa were represented at significantly lower levels, ranging from 0.85 (*B. subtilis*) down to 0.0037% (*Staphylococcus aureus*).

Collectively, these results show that Sequenoscope can successfully process both long-read ONT and short-read Illumina data, with observed relative abundances remaining close to expected values, confirming its utility for cross-platform analysis.

## Conclusion

This article introduces Sequenoscope, a modular pipeline primarily designed for the comparison of different test and control ONT AS enrichment and depletion conditions. We used two ZymoBIOMICS Microbial Community DNA Standards with ONT’s AS depletion of *L. monocytogenes* to demonstrate the capabilities and wide application potential of Sequenoscope. The pipeline successfully handles short-read datasets from the Illumina platform in addition to its core ONT long-read platform support, establishing itself as a versatile cross-platform solution for both standard and AS comparative metagenomic analyses. The primary goal of our research is to facilitate the adoption of ONT sequencing using AS by providing an accessible and efficient analytical framework, allowing users to quickly and conveniently assess such experimental runs. Through Sequenoscope, we aim to simplify the post-run analytics and quality control aspects of ONT AS, making this advanced technique more approachable and feasible for researchers. The significance of our findings underscores the practicality of combining AS data with an intuitive analytical pipeline, thereby promoting wider adoption of AS in genomics. The integration of user-friendly tools with modern NGS technologies is crucial to enhance the accessibility, applicability and interpretability of advanced genomic sequencing techniques, contributing to a deeper understanding and exploration of genomics and metagenomics.

## Abbreviations

AS: adaptive sampling
NGS: next-generation sequencing
ONT: Oxford Nanopore Technologies
